# SARS-CoV-2 Inhibition by Sulfonated Compounds

**DOI:** 10.3390/microorganisms8121894

**Published:** 2020-11-30

**Authors:** Matteo Gasbarri, Philip V’kovski, Giulia Torriani, Volker Thiel, Francesco Stellacci, Caroline Tapparel, Valeria Cagno

**Affiliations:** 1 Institute of Materials, Ecole Polytechnique Fédérale de Lausanne, 1015 Lausanne, Switzerland; matteo.gasbarri@epfl.ch (M.G.); francesco.stellacci@epfl.ch (F.S.); 2 Institute of Virology and Immunology (IVI), Länggassstrasse 122, 3012 Bern, Switzerland; philip.vkovski@ifik.unibe.ch (P.V.); volker.thiel@vetsuisse.unibe.ch (V.T.); 3 Institute for Infectious Diseases, University of Bern, Hochschulstrasse 6, 3012 Bern, Switzerland; 4 Department of Microbiology and Molecular Medicine, University of Geneva, 1211 Geneve, Switzerland; giulia.torriani@unige.ch (G.T.); caroline.tapparel@unige.ch (C.T.); 5 Institute of Bioengineering, Ecole Polytechnique Fédérale de Lausanne, 1015 Lausanne, Switzerland

**Keywords:** SARS-CoV-2, antiviral, heparan sulfates, attachment inhibitor

## Abstract

Severe acute respiratory syndrome-related coronavirus 2 (SARS-CoV-2) depends on angiotensin converting enzyme 2 (ACE2) for cellular entry, but it might also rely on attachment receptors such as heparan sulfates. Several groups have recently demonstrated an affinity of the SARS-CoV2 spike protein for heparan sulfates and a reduced binding to cells in the presence of heparin or heparinase treatment. Here, we investigated the inhibitory activity of several sulfated and sulfonated molecules, which prevent interaction with heparan sulfates, against vesicular stomatitis virus (VSV)-pseudotyped-SARS-CoV-2 and the authentic SARS-CoV-2. Sulfonated cyclodextrins and nanoparticles that have recently shown broad-spectrum non-toxic virucidal activity against many heparan sulfates binding viruses showed inhibitory activity in the micromolar and nanomolar ranges, respectively. In stark contrast with the mechanisms that these compounds present for these other viruses, the inhibition against SARS-CoV-2 was found to be simply reversible.

## 1. Introduction

Severe acute respiratory syndrome-related coronavirus 2 (SARS-CoV-2) is causing an unprecedented pandemic, and a better understanding of its biology and pathogenesis is required to identify effective antiviral strategies.

Presently, the only approved antiviral drug is remdesivir, a nucleoside analogue that inhibits viral RNA synthesis of several coronaviruses [1]. However, the results of randomized double-blind clinical trials showed only a reduction in hospitalization time (from 15 to 11 days), while mortality was not significantly reduced compared to placebo [2,3]. Hydroxychloroquine, despite being widely used, did not show any activity in human respiratory cell lines [4], animal models [5], and in vivo clinical trials [6,7]. Conversely, dexamethasone, a corticosteroid, significantly reduced mortality in patients with severe COVID-19 [8]; the efficacy of tocilizumab, a monoclonal antibody directed against interleukin-6 receptor, is being evaluated in clinical trials [9], and anticoagulant treatments have proven to be beneficial [10], demonstrating that targeting the excessive activation of the immune response is a promising strategy.

However, inhibiting viral replication during the initial stages of infection rather than treating the symptoms resulting from immune activation and inflammation will be largely more beneficial in preventing hospitalization and long-term sequelae of infection. For this reason, development of direct-acting antiviral compounds should be a research priority. One class of antiviral compounds in study are attachment inhibitors, with a strategy that is intrinsically broad-spectrum, since many different viruses use similar attachment receptors.

The two major classes of attachment receptors used by viruses are heparan sulfates (HS) [11] and sialic acid [12]. These receptors are widely expressed on eukaryotic cells and used by a wide range of viruses to adhere to the cell surface before interacting with a more specific entry receptor, which triggers uptake and entry [11]. This strategy is used as well by coronaviruses (CoVs). Middle East Respiratory Syndrome (MERS)—CoV uses sialic acid (with a preference for α2,3-linked SAs) [13], while NL63 [14] and Severe Acute Respiratory Syndrome (SARS) CoV [15] were reported to use HS. In addition to adhesion receptors, NL63, SARS, and SARS-CoV-2 use angiotensin converting enzyme 2 (ACE2) as an entry receptor [16,17].

Sialic acid dependency of SARS-CoV-2 has so far only been investigated with computational methods [18,19], and no biological confirmation is available to date. In contrast, given the sequence similarity between SARS-CoV-2 and SARS-CoV, the dependence on HS has already been investigated by many groups with different approaches. Several reports have shown that HS can act as a co-receptor for SARS-CoV2, inducing a conformational change on the spike that enhances the interaction with ACE2 [20,21,22].

In the work of Kim et al., the interaction of the receptor binding domain of the spike (S) protein of SARS-CoV-2 with heparin and HS was shown by surface plasmon resonance (SPR) [21]. Recombinant S protein was also used in glycan array studies to evaluate the interaction with different glycans, and highlighted a higher binding for highly sulfated glycans [23]. In two recent studies [20,22], the ability of heparin or heparinase treatment to inhibit the binding of SARS-CoV-2 was shown both with pseudotyped or wild-type viruses. In addition, docking studies [20] suggest that the interaction between S and HS is mediated by a site in proximity to but independent from the ACE2 binding domain. Interestingly, Clausen et al. [20] also pointed out that the virus is less dependent on HS on Vero E6, due to a high expression of ACE2.

The proposition of using heparin and heparin analogues to treat COVID-19 is extensively discussed by Tiwari et al. [24], and others are proposing to repurpose drugs mimicking or targeting HS, such as the anticancer pixatimod (PG454) [25], or mitoxantrone, a drug used for acute nonlymphocytic leukemia, prostate cancer, and multiple sclerosis [26].

Additionally, unfractionated heparin (UFH) and low-molecular-weight heparin (LMWH) are being tested in clinical trials. Heparin is known to have anti-clotting, anti-coagulant, anti-thrombotic, and anti-inflammatory properties and could therefore be effective in treating the coagulopathy and hyper inflammatory response characteristic of critically ill COVID-19 patients. However, this use is unrelated to the direct antiviral activity exerted on viral attachment [27].

Based on these published studies, we tested sulfonated compounds that were previously shown to be active against HS-dependent viruses. In the past, we synthetized gold nanoparticles [28], and β-cyclodextrins [29] coated with mercapto-undecan-sulfonates. In contrast to heparin and other molecules, in which a long alkyl moiety is not present, our compounds were endowed with an irreversible mechanism of action in the absence of toxicity. Upon interaction with HS-dependent viruses, the multivalent binding coupled to structural features of our compounds led to structural damage of the viruses, i.e., the compounds displayed a virucidal activity.

Here, we show that sulfonated materials with a hydrophobic component show inhibitory activity in the same concentration range reported for HS-dependent viruses, but unlike what was observed for a number of other viruses, the inhibition for SARS-CoV-2 is virustatic (reversible) and not virucidal (irreversible).

## 2. Materials and Methods

### 2.1. Compounds and Synthesis of Materials

Heparin sodium salt from porcine mucosa (H4784), Enoxaparin Sodium US Pharmacopeia (USP) Reference Standard (1235820), European Pharmacopoeia (EP) Reference standard, ι-carrageenan and β-cyclodextrins sulfated sodium salt (CAS: 37191-69-8) were purchased from Sigma. K5N,OS(H) was obtained by Glycores SRL. Resonium A was purchased from Sanofi. Hydroxychloroquine and Heparin sodium salt (2812) were purchased from Tocris bioscience.

MUS:OT NP and MUS-CD were synthetized as previously described [28,29], and the characterization is shown in Figures S1 and S2.

### 2.2. Cells and Virus

Vero C1008 (clone E6) (ATCC CRL-1586) cells were a kind gift from Prof Gary Kobinger, and were propagated in DMEM High Glucose + Glutamax supplemented with 10% fetal bovine serum (FBS) and 1% penicillin/streptavidin (pen/strep).

SARS-CoV2/Switzerland/GE9586/2020 was isolated from a clinical specimen in the University Hospital in Geneva in Vero-E6 and passaged twice before the experiments. SARS-CoV-2/München-1.1/2020/929 (kindly provided by M. Müller and C. Drosten; Charité, Berlin, Germany) was propagated on Vero-E6 cells cultured in Dulbecco’s modified minimal essential medium supplemented with 10% heat inactivated fetal bovine serum, 1% non-essential amino acids, 100 µg/mL of streptomycin, 100 IU/mL of penicillin, and 15 mM of HEPES. Supernatant of infected cells was collected 3 days post infection, clarified, aliquoted, and frozen at −80 °C and subsequently titrated by plaque assay in Vero-E6.

### 2.3. VSV-CoV-2 Production

Vesicular stomatitis virus (VSV)-based SARS-CoV-2 pseudotypes (VSV-CoV-2) generated according to [30] and [31] expressing a 19 amino acids C-terminal truncated spike protein (NCBI Reference sequence:NC_045512.2) were produced in HEK293F and titrated in Vero-E6.

### 2.4. VSV-CoV-2 Inhibition Assays

Vero-E6 cells (13,000 cells per well) were seeded in a 96-well plate. Compounds were serially diluted in DMEM and incubated with VSV-CoV-2 (MOI, 0.001 ffu/cell) for 1h at 37 °C. The mixture was added on cells for 1h at 37 °C. The monolayers were then washed and overlaid with medium containing 2% FBS for 18h. The following day cells were fixed with paraformaldehyde 4%, stained with DAPI, and visualized using an ImageXpress Micro XL (Molecular Devices, San Jose, CA, USA) microplate reader and a 10× S Fluor objective. The percentage of infected cells was estimated by counting the number of cells expressing GFP and the total number of cells (DAPI-positive cells) from four different fields per sample using MetaXpress software (Molecular Devices, San Jose, CA, USA).

### 2.5. Plaque Assay on VERO-E6 Cells

Vero-E6 cells (100,000 cells per well) were seeded in a 24-well plate. Compounds were serially diluted in DMEM and incubated with SARS-CoV-2 (MOI, 0.005 PFU/cell) for 1 h at 37 °C. The mixture was added on cells for 1h at 37 °C. The monolayers were then washed and overlaid with 0.8% avicel rc581 in DMEM supplemented with 5% FBS. For the experiments described in Figure S3, the protocol is the same as described with two modifications: the number of cells seeded is 83,000 cells per well and the overlay contained 1.2% avicel. Two days after infection, cells were fixed with paraformaldehyde 4% and stained with crystal violet solution containing ethanol. Plaques were counted, the percent inhibition of virus infectivity was determined by comparing the number of plaques in treated wells with the number in untreated control wells, and 50% effective concentration (EC_50_) was calculated with Prism 8 (GraphPad, San Diego, CA, USA). Serial dilutions of hydroxychloroquine were added on cells 1h before infection and readded during infection.

### 2.6. Virucidal Assay

Viruses (10^5^ pfu of SARS-CoV-2) and or MUS:OT NPs or MUS CD (300 µg/mL) were incubated for 1 h at room temperature, and then the virucidal effect was investigated by adding serial dilutions of the mixtures on Vero-E6 for 1h, followed by addition of medium containing avicel as described above. Viral titers were determined at dilutions at which the material was not effective.

## 3. Results

**Figure 1:**
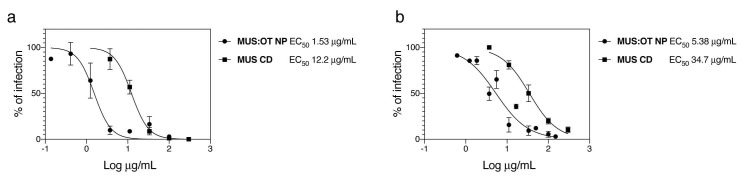
Inhibitory activity of sulfonated nanomaterial against severe acute respiratory syndrome-related coronavirus 2 (SARS-CoV2). (**a**) Vesicular stomatitis virus (VSV)-CoV-2 or (**b**) SARS-CoV-2 were incubated for 1 h at 37 °C with different doses of MUS:OT NP or MUS CD and subsequently serially added on cells. In (**a**), the number of GFP positive cells was evaluated 24 hpi while in (**b**) the number of plaques was determined 48 hpi. Results are expressed as mean and SEM of three independent experiments.

In order to evaluate the ability of sulfonated compounds to inhibit the attachment of SARS-CoV-2, we pre-incubated VSV pseudo-viruses expressing the spike protein of SARS-CoV-2 (VSV-CoV-2) or SARS-CoV-2 wild-type viruses and the compounds for 1 h at 37 °C, followed by addition on cells. After 1 h, cells were washed and the infection was quantified 48 hpi by plaque assay for the wild-type virus or 24 hpi through GFP-positive cells quantification for VSV-CoV-2. The results (Figure 1) show that our nanoparticles (MUS:OT NP) and cyclodextrins (MUS CD) showed inhibitory activity in both cases.

Importantly, the results on SARS-CoV-2 with MUS:OT NP and MUS CD were confirmed in two independent labs with two different strains of SARS-CoV-2 (SARS-CoV-2/München-1.1/2020/929 and SARS-CoV2/Switzerland/GE9586/2020). In both cases, MUS:OT NP showed higher potency than MUS CD (Figure 1 and Figure S3).

**Figure 2:**
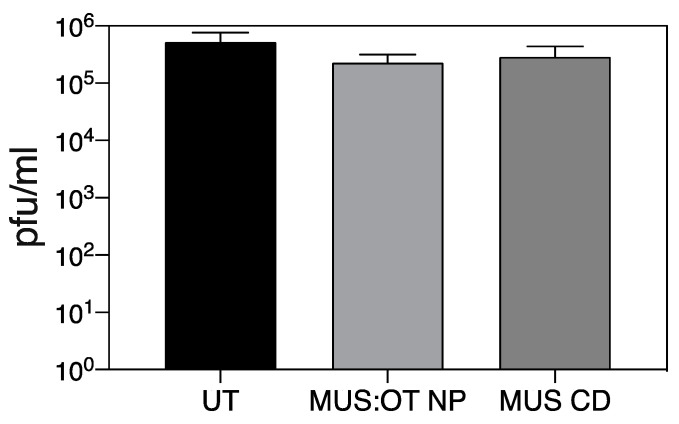
Virucidal activity. 10^5^ pfu of SARS-CoV-2 was incubated for 1 h at 37 °C with 300 µg/mL and subsequently serially diluted on cells. Infectious titers were evaluated for each treatment condition at dilutions at which the concentration of compound was not active. Results are expressed as mean and SEM of three independent experiments.

The two compounds previously showed virucidal activity against different HS-dependent viruses, i.e., the ability to permanently impair viral infectivity. Therefore, we assessed whether they also displayed virucidal activity against SARS-CoV-2. SARS-CoV-2 (10^5^ pfu) was incubated for 1h with 300 µg/mL of the nanomaterials and the mixture was subsequently diluted on cells. The residual infectivity was evaluated at concentrations of molecules known to be non-inhibitory. The results (Figure 2) evidence a lack of virucidal activity of both materials.

To address the HS-dependency of our SARS-CoV-2 strains, we tested, with the same protocol described for MUS:OT NP and MUS CD, the antiviral effect of heparins from different sources, of another sulfated polymer (K5N,OS(H)) and of commercially available sulfated beta-cyclodextrins against VSV-CoV-2 and SARS-CoV-2. All of these molecules failed to show antiviral activity up to 1000 µg/mL, while a sulfated polymer, carrageenan, showed very weak antiviral activity but only against wild-type SARS-CoV-2 (Table 1).

## 4. Discussion

Here, we show the antiviral activity of sulfonated compounds against SARS-CoV-2. These compounds (MUS:OT NP and MUS CD) were previously reported to exert a virucidal (i.e., irreversible) activity against HS-dependent viruses such as Herpes Simplex Virus 2, respiratory syncytial virus, papillomavirus, and dengue virus [28,29], and virustatic (i.e., reversible) activity against vesicular stomatitis virus (VSV), an HS-independent virus. The results displayed in Figure 1 and Figure 2 show reversible inhibition for both compounds against SARS-CoV-2, similarly to what we previously reported for VSV. In addition, we show that other HS-mimicking compounds are inactive against SARS-CoV2, as also reported for VSV.

Two different scenarios could account for the lack of virucidal activity of MUS:OT NP and MUS CD against SARS-CoV-2. The restricted virustatic effect may be explained by the peculiar shape of CoVs, whose receptor binding domain (RBD) is approximately ten nanometers away from the viral envelope. We suggested that the virucidal activity of our compound results from a pressure exerted on the RBD of the viral glycoprotein that is then transmitted to the whole virion [28]. This mechanism may then not be applicable to CoVs due to the distance between the RBD and the envelope. Alternatively, and as previously described for VSV [32], the absence of virucidal activity of MUS:OT NP and MUS CD could be due to the poor affinity of the SARS-CoV-2 spike protein for HS. This second scenario is supported by the absence of inhibitory activity of heparin and other sulfated compounds in our experimental settings. Of note, the observed virustatic effect of our compound compared to the absence of activity of various types of sulfated compounds could be explained by the presence of long hydrophobic linkers that may enhance binding to the basic amino-acid residues of the spike protein. We acknowledge that our results suggest that HS is not used by SARS-CoV2 for infection, and there is abundant literature showing the opposite [20,22,23,26,27,33]. However, also in the current literature, discrepancies in the antiviral potencies of the heparins and heparin analogues are present [33]. For instance, Tandon et al. report IC_50_ of 5.99 μg/L, 1.08 mg/L, for UFH and enoxaparin, respectively, while Tree et al. [27] report values of 41 μg/mL and 7800 μg/mL, respectively. In our experimental setting, both UFH and LMWH do not show efficacy up to 1000 μg/mL.

The discrepancy of our data with the existing literature could be explained by different hypotheses: (i) differences in the clinical isolate used: point mutations in the spike protein might result in different binding of the virus to HS, as reported for other viruses [34]; moreover, it is well known that multiple passaging in cell culture could lead to cell adaptation and the acquisition of the ability to bind heparan sulfates [11]. In order to prevent this from happening, our clinical isolates were passaged only twice in cells before the experiment, and there is no information about viral passage number in some of the published reports [20,22]. (ii) The secondary role of HS in SARS-CoV-2 entry and the presence of distinct putative domains of interaction on the spike protein of SARS-CoV-2 for HS and ACE2. Indeed, Clausen et al. showed that SARS-CoV-2 exploits ACE2 as primary receptor for cell recognition, using heparan sulfate (HS) only as a binding enhancer. They also reported that in Vero-E6, the cell line used in our study, the abundance of ACE2 decreases the dependency of the virus on HS.

## 5. Conclusions

In conclusion, we show that sulfonated cyclodextrin and nanoparticle show, respectively, micromolar and nanomolar inhibitory activity against SARS-CoV-2, further broadening the number of viruses inhibited by these compounds. However, the reversible nature of the inhibition points to less clinical relevance. The lack of irreversible activity could be due to the peculiar shape of coronaviruses or to the low dependency of SARS-CoV-2 on HS for viral attachment.

## Figures and Tables

**Table 1:** Antiviral activity of heparan sulphates (HS) mimicking compounds against VSV-CoV-2 and SARS-CoV-2.

	Compound	EC_50_ (µg/mL)	EC_50_ µM	CC_50_ (µg/mL)
**VSV-CoV-2**	Carrageenan	>300	>317	>300
	K5N,OS(H)	>300	>20	>300
	Sulfated ß-CD	>300	>145	>300
	Resonium A	>300	>300	>300
	MUS:OT NP	1.5	0.005	>300
	MUS-CD	12.2	4.0	>300
	Hydroxychloroquine		3.9	-
**SARS-CoV-2**	Carrageenan	267	282	>300
	K5N,OS(H)	>300	>20	>300
	Sulfated ß-CD	>1000	>485	>300
	MUS:OT NP	5.38	0.017	>300
	MUS-CD	35.0	11.5	>300
	Heparin sodium salt (Tocris)	>1000	>40	
	Heparin Sodium Salt (Sigma)	>1000	>55	>300
	Enoxaparin Sodium Salt	>1000	>200	
	Hydroxychloroquine		2.92	-

EC50 50% effective concentration, CC50 50% cytotoxic concentration.

## Supplementary Materials

The following are available online at https://www.mdpi.com/2076-2607/8/12/1894/s1. Figure S1. Characterization of MUS: OT NPs. Figure S2. Characterization of MUS CD. Figure S3. Inhibitory activity of sulfonated nanomaterial against SARS-CoV2.
