# Supplementary material for: SARS-CoV-2 Inhibition by Sulfonated Compounds
Source: Microorganisms. 2020 Nov 30;8(12):1894. doi: 10.3390/microorganisms8121894 (PMC7760145; doi:10.3390/microorganisms8121894)
Supplement: Supplementary file 1 [file microorganisms-08-01894-s001.pdf]

a) 1H-NMR	b) TEM image	c) Size distribution
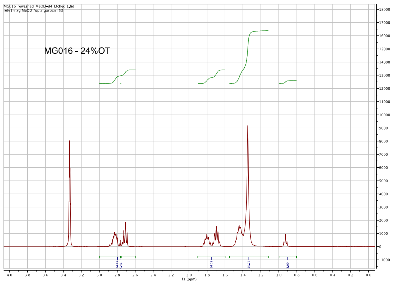	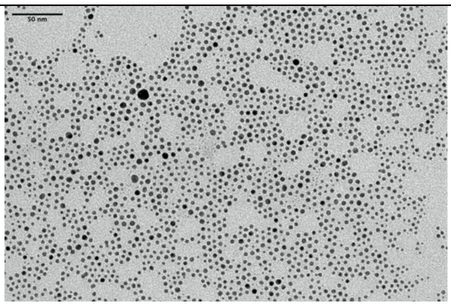	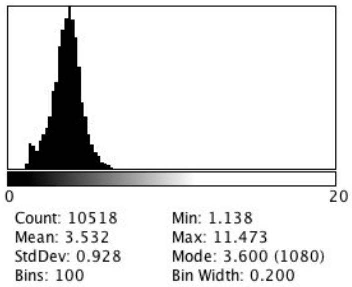

Figure S1. Characterization of MUS:OT NPs. (a) Ratio between MUS:OT was calculated by 1H-NMR (400 MHz, Bruker AVANCE 400). The ratio between MUS:OT was determined to be 24% OT and 76% MUS. (b) TEM image of AuNPs taken by Tecnai Osiris Size of the core was evaluated using a ImageJ plug-in analyzing more than 10000 particles. (c) The diameter of the core is 3.5 ± 1 nm. MW can be estimated to be around 320kDa.

a)

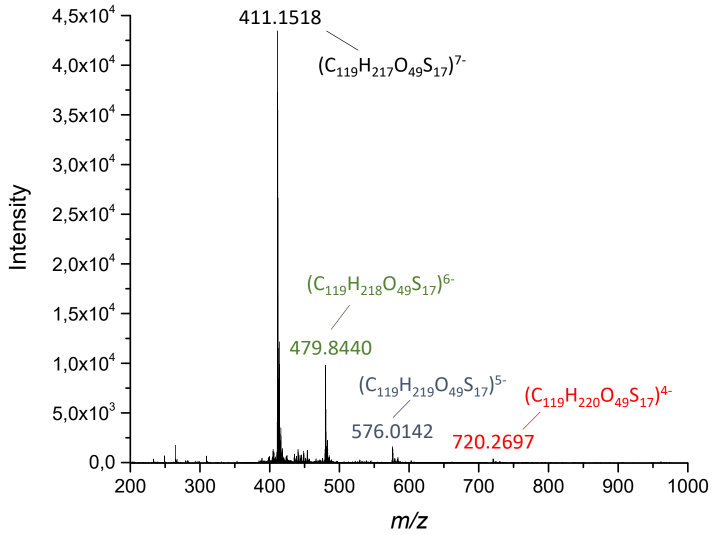

b)

Molecular Formula	Theoretical (Da)	Experimental (Da)	Error (ppm)
(C119H217O49S17)7-	411.1518	411.1523	1.6
(C119H218O49S17)6-	479.8448	479.8440	1.7
(C119H219O49S17)5-	576.0152	576.0142	1.9
(C119H220O49S17)4-	720.2709	720.2697	1.6
(C119H221O49S17)3-	960.6969	960.6948	2.2

c)

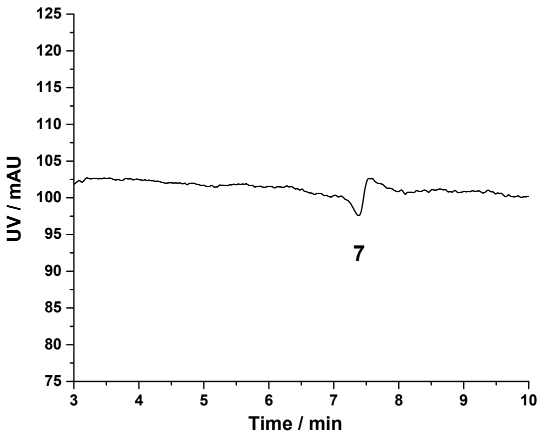

Figure S2. Characterization of MUS CD. (a) HRMS Cold-Spray Ionisation spectrum of MUS-CD in EtOH/H2O (50:50), (b) HRMS Summary of MUS-CD in EtOH/H2O (50:50). Values correspond to the monoisotopic mass, (c) CE-UV electropherogram of MUS-CD at 230 nm with assigned CD species showing 100% of 7 sulfonates per CD.

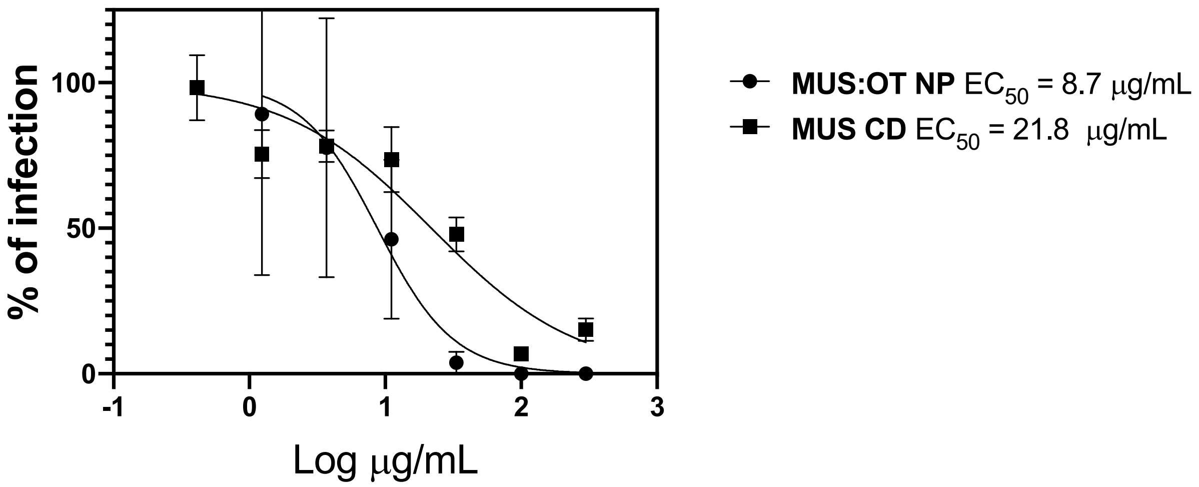

Figure S3. Inhibitory activity of sulfonated nanomaterial against SARS-CoV2. SARS-CoV-2 was incubated for 1h at 37°C with different doses of MUS:OT and MUS CD and subsequently serially added on cells. Number of plaques was determined 48hpi. Results are expressed as mean and SEM of two independent experiments.
